# Looking Back at the 2018 Year of *Stem Cell Reviews and Reports*

**DOI:** 10.1007/s12015-018-9859-0

**Published:** 2018-10-18

**Authors:** Mariusz Z. Ratajczak

**Affiliations:** University of Louisville, Louisville, KY USA

The second year of *SCRR* under new leadership closes with this December 2018 issue and we are pleased to announce that impact factor increased to 3.612. We published almost 80 articles this year, which were carefully evaluated by me and five highly respected section editors: Drs. Giovanni Camussi, Henning Ulrich, Louis Pelus, Peter Quesenberry and Edward Scott, and as well as 17 editorial board members. This scholarly output was also critically dependent on our dedicated reviewers, and we are grateful to them for their hard work. *Stem Cell Reviews and Reports* covers a broad range of topics, including different aspects of stem cell biology and tissue/organ regeneration. We are open not only to clear scientific progress but also to new and challenging ideas as well as some controversies in the field - following a famous quote of Albert Einstein who used to say that “*A blind belief in authority is worst enemy of truth*”.

The scientific stem cell community is on a search to identify a pluripotent stem cell able to differentiate into cells from all three germ layers: meso-, endo- and ectoderm that could be safely employed in the clinic. It was a great expectation that embryonic stem cells (ESCs) or induced pluripotent stem cells (iPSCs) will rapidly fulfill this mission.

Unfortunately, the use of ESCs has not been only ethically controversial but more important they have technical problems, such as the risk of teratoma formation, potential histoincompatibility with unrelated recipients and genomic instability. In response to these problems, a solution for obtaining ethically acceptable PSCs has been proposed: generating induced pluripotent stem cells (iPSCs) by genetic modification of adult cells. However, these cells have also been found to be at risk of teratoma formation and immunological rejection and - what flashes a bright red light - data accumulates demonstrating their genomic instability. Moreover, the current results for clinical applications of iPSCs have demonstrated only paracrine effects in therapy and no contribution of these cells to damaged organs. This has been recently discussed by Dr. Bhartiya [1]. This all suggests a risk of an approaching twilight for the clinical application of ESCs and iPSCs, unless some strategies will be developed to avoid these limitations. Nevertheless, our journal is open to papers discussing potential application of iPSCs and two interesting reports have been published. In the first Dr. Binah’s group reviews the latest studies combining iPSC and CRISPR/Cas9 technologies for the investigation of the molecular and cellular mechanisms underlying inherited diseases including immunological, metabolic, hematological, neurodegenerative and cardiac diseases [2]. In the second Dr. Slukvin et al. report advances in the chemical modifications of messenger RNA as an alternative nucleic acid-based transgene-free approach for scalable production of iPSCs for drug screening and therapeutic purposes. These findings provide valuable information on the design of in vitro transcription templates being used in PSCs and its broad applicability for basic research, disease modelling, and regenerative medicine [3].

In parallel a major focus of our journal are potential pluri/multipotent stem cells isolated from the adult tissues including a population of small, early-development stem cells that express pluripotency markers and that, based on their primitive morphology and gene expression profile, named very small embryonic-like stem cells (VSELs). In the past year few interesting papers have been published on this topic showing an efficient ex vivo expansion of these small cells in a presence of UM177 reported in August issue by Dr. Henon’s group as well as demonstration by Dr. Virant-Klun that ovarian surface epithelium VSELs derived oocyte-like cells respond to sperm cells by releasing of zona pelucida [4]. These observations confirm the approaching possibility to potentially employ these cells in the clinic and a presence of VSELs-like germinal stem cells in the ovarian surface epithelium.

One of the challenges in stem cell research is better understanding of molecular mechanisms involved in regulating stem cell pluripotency and differentiation. Evidence accumulated that specific miRNAs control cell cycle associated molecules and checkpoints in embryonic, somatic and cancer stem cells [5]. In another interesting paper published in February issue Dr. Zakian’s group discusses involvement of non-coding mRNA as substantial components of regulatory networks in early development stem cells.

In a short editorial it is not possible to summarize all of the papers that were published in our journal over the past year. We encourage our readers to have a close look at the papers highlighted above as well as other outstanding publications. Going forward, *Stem Cell Reviews and Reports* will continue to publish the latest discoveries and to entertain challenging and provocative ideas. We encourage you to submit your best work and help establish our journal as a premier journal in this important field.
